# Supplementary material for: Recurrent gain of function mutation in calcium channel CACNA1H causes early-onset hypertension with primary aldosteronism
Source: eLife. 2015 Apr 24;4:e06315. doi: 10.7554/eLife.06315 (PMC4408447; doi:10.7554/eLife.06315)
Supplement: Supplementary file 1. — (A) Clinical features of 40 patients with primary aldosteronism. (B) Sequencing statistics of 40 exomes. (C) Previously unreported protein-altering variants that occur in more than one subject. (D) Genes with highest burden of rare (<0.01%) heterozygous variants in cases compared to controls. (E) Genes with highest burden of rare (<0.1%) homozygous, hemizygous or candidate compound heterozygous damaging or conserved variants in cases compared to controls. (F) Illumina sequence reads identifying CACNA1H p.Met1549Val in five unrelated subjects. (G) Demonstration of biological parentage by genotyping of short tandem repeat markers in parent-offspring trios in kindreds 1347 and 1390 confirms that CACNA1H mutations are de novo in these kindreds. (H) Kinship coefficients of affected individuals from kindreds with CACNA1HM1549V variant. (I) Clinical features of family members of index cases with CACNA1HM1549V. DOI: http://dx.doi.org/10.7554/eLife.06315.017 [file elife06315s004.docx]

**Supplementary file 1**

**Supplementary file 1A.**

**Clinical features of 40 patients with primary aldosteronism**

| **ID** | **Gender** | **Ethnicity** | **Age dx** | **BP dx** | **K^+^ dx** | **Age referral** | **BP referral** | **K^+^ referral** | **Aldo** | **PRA** | **ARR** | **Therapy for hyperaldosteronism** |
| --- | --- | --- | --- | --- | --- | --- | --- | --- | --- | --- | --- | --- |
| 110-1 | M | CEU | 6 | NA | NA | 6 | 144/88 | 3.3 | 45.9 | 2 | 23 | none |
| 113-1 | M | CEU | 9 | NA | 2.7 | 10 | 139/85 | NA | 29 | <0.2 | >145.0 | CAP, SPR |
| 247-1 | F | AA | 9 | NA | NA | 22 | 134/96 | 3.2 | NA | NA | >30.0* | KCl, LIS |
| 270-2 | F | CEU | 7.5 | NA | NA | 9 | 129/96 | 2.9 | 18 | 0.42 | 42.9 | SPR |
| 318-1 | M | CEU | 7 | 170/140 | NA | 13 | 130/90 | 2.6 | 9.5 | 0.21 | 45.2 | SPR, NIF |
| 333-2 | M | CEU | 9 | 192/144 | 5.5 | 17 | 150/92 | 4.1 | 40 | <0.7 | >57.0 | SPR, MIN, ATN, HCTZ, LIS |
| 461-1 | M | AA | 0.16 | 120/70 | NA | 0.25 | 96/59 | 5.5 | 195 | 0.4 | 487.5 | HDR, SPR, FUR |
| 531-1 | F | CEU | 6 | NA | NA | 20 | 280/188 | NA | 100 | <0.3 | > 333.3 | NA |
| 712-1 | F | HIS | 6 | 162/90 | NA | 9 | 127/79 | 2.2 | 42 | 0.8 | 52.5 | AMIL, KCl, NIF |
| 771-1 | F | CEU | 0.25 | 161/81 | NA | 0.42 | 90/56 | 5.5 | 40 | <0.1 | >400.0 | DEX |
| 950-1 | M | AA/CEU | 9 | 180/120 | NA | 9 | 150/68 | 3.7 | 38.3 | 0.2 | 191.5 | ENL, AMLD |
| 1019-1 | M | non-CEU | 8 | 140/100 | 4.4 | 10 | 148/100 | NA | 41 | 1.8 | 22.8 | ENL |
| 1024-1 | M | AA/CEU | 0.08 | 130/80 | nL | 0.42 | 90/50 | NA | 77 | 0.18 | 427.8 | SPR |
| 1086-1 | F | CEU | 0.17 | 160/100 | NA | 1 | 96/NA | 4.1 | 98 | <0.2 | >490.0 | LAB |
| 1105-1 | M | HIS | 9 | 150/95 | 1.9 | 10 | 100/60 | NA | 45.5 | 1.1 | 41.4 | SPR, αMD, CAN |
| 1108-1 | F | HIS | 4 | 142/94 | 4 | 5 | 108/60 | NA | 18 | <0.2 | >90.0 | CAP, AMLD, CTZ |
| 1147-1 | M | CEU | 2 | 161/95 | NA | 2 | 106/64 | 5.3 | 45 | 0.1 | 450 | AMLD |
| 1228-2 | F | HIS | 0.13 | 120/85 | 4.8 | 0.13 | 140/100 | NA | 120 | <0.2 | >600.0 | LAB, HDR |
| 1281-1 | F | AA | NA | NA | low | 1.5 | 117/71 | 4.1 | 17 | <0.5 | >34.0 | AMLD |
| 1283-1 | F | CEU | 0 | NA | NA | 2 | 106/44 | 4.6 | 55 | 0.6 | 91.7 | PRZ, CLO, SPR, AMLD |
| 1301-1 | M | CEU | 0.17 | 128/80 | NA | 0.33 | 100/60 | 3 | 52 | <0.2 | >260.0 | AMLD, SPR |
| 1325-1 | M | AA | 0.25 | 122/84 | low | 0.5 | 120/90 | 5.9 | 112.8 | 0.1 | 1128 | PRP, DEX, HDR, SPR, FUR |
| 1342-1 | M | CEU | 0.08 | 120/80 | 5.1 | 0.08 | 120/70 | NA | 66.4 | 0.55 | 120.7 | SPR, HCTZ, NP |
| 1347-1 | M | CEU | 3 | 160/105 | 3.8 | 7 | 145/71 | NA | 20 | <0.1 | >200.0 | ISR, SPR |
| 1368-1 | M | CEU | 8 | 140/90 | 3.9 | 14 | 118/74 | 4.2 | 20 | <0.2 | >100.0 | LIS, AMLD |
| 1381-1 | F | CEU | 7 | NA | NA | 7 | 135/94 | 3.5 | 34 | <0.1 | >340.0 | none |
| 1390-1 | F | AA | 7 | 150/90 | NA | 10 | 145/88 | 3.1 | 66 | 0.2 | 330.0 | HCTZ, LIS |
| 1393-1 | M | HIS | 0.17 | 170/110 | NA | 0.25 | NA | 4.1 | 87.2 | <0.6 | >145.3 | PRP, CAP, HDR, SPR |
| 1431-1 | F | CEU | 0.15 | 100/60 | 5.9 | 0.17 | 75/59 | NA | 45.6 | 0.6 | 76 | ISR, LAB, NIF |
| 1450-1 | M | AA | 0 | 119/74 | NA | 0.3 | 133/82 | 4.7 | 55.8 | 0.1 | 558 | AMLD, AMIL, ENL, αMD |
| 1452-1 | M | CEU | 0.21 | 110/70 | NA | 0.56 | 132/NA | NA | 58.6 | 0.1 | 586 | HDR |
| 1472-1 | F | CEU | 0.25 | NA | NA | 1 | 93/54 | 5.1 | 50 | <0.6 | >83.3 | SPR |
| 1490-1 | M | CEU | 0.16 | 140/NA | NA | 0.25 | NA | NA | 61.1 | 0.2 | 305.5 | HDR, AMIL, SPR |
| 1492-1 | M | CEU | 0.16 | 150/90 | 4 | 0.25 | 123/90 | NA | 63.8 | <0.15 | >425.3 | CAP |
| 1506-1 | F | PR | 5 | 151/95 | 3.3 | 5 | 115/73 | 4.3 | 14 | <0.6 | >23.3 | AMIL, DEX, SPR, HC |
| 1512-1 | F | CEU | 10.5 | 148/81 | 4.2 | 10 | 120/70 | NA | 24.5 | <0.3 | >81.7 | SPR |
| 1524-1 | F | CEU | 9 | 177/128 | 3.7 | 9 | 130/90 | NA | 19.7 | 0.19 | 103.7 | LIS, EPL |
| 1543-1 | M | CEU | 0 | NA | 3.7 | 0.5 | 92/60 | 4.1 | >72.2 | <0.04 | >1805 | SPR |
| 1578-1 | M | CEU/AI | 0 | 110/61 | NA | 0.58 | 102/- | 4.8 | 22.9 | 0.4 | 57.3 | HDR, ISR |
| 1600-1 | M | CEU | 0.08 | 95/55 | 6.3 | 0.08 | 80/41 | 5.3 | 111 | 0.1 | 1110 | HDR |

M, male; F, female; CEU, of European origin; AA, of African American origin; HIS, of Hispanic origin; AI, of American Indian origin; PR, Puerto Rico. Mixed origin is indicated by “/”; Age dx/referral, age at diagnosis of hypertension/referral; BP dx/referral, blood pressure at diagnosis/referral; K^+^ dx/referral, serum potassium (mmol/L, normal 3.5-5.5) at diagnosis/referral; Aldo, serum aldosterone (ng/dL); PRA, plasma renin activity (ng/mL/h); ARR, aldosterone:renin ratio (ng/dL:ng/mL/h, values >20 with aldosterone level greater than 15 are considered indicative of primary aldosteronism); NA, not available; nL, within normal limits; *documented ARR without available PRA and aldosterone values. CAP, captopril; SPR, spironolactone; LIS, lisinopril; NIF, nifedipine; MIN, minoxidil; ATN, atenolol; HCTZ, hydrochlorothiazide; HDR, hydralazine; FUR, furosemide; AMIL, amiloride; DEX, dexamethasone; ENL, enalapril; CTZ, chlorothiazide; AMLD, amlodipine; LAB, labetalol; αMD, alpha-methyldopa; CAN, candesartan; PRZ, prazosin; CLN, clonidine; PRP, propranolol; ISR, isradipine; HC, hydrocortisone; EPL, eplerenone. Subjects with the *CACNA1H^M1549V^* mutation are shown in red.

**Supplementary file 1B.**

**Sequencing statistics of 40 exomes**

| **Mean independent reads per targeted base** | 72.7 |
| --- | --- |
| **Median independent reads per targeted base** | 61.5 |
| **% of bases mapping to genome** | 92.0 |
| **% of bases mapping to exome** | 67.1 |
| **% of targeted bases with ≥ 8 independent reads** | 95.0 |
| **Mean error rate (%)** | 0.4 |

**Supplementary file 1C.**

**Previously unreported protein-altering variants that occur in more than one subject**

| **Gene** | **# of independent instances among 40 cases** | **# of *de novo* variants** | **p-value** | **Expression in top 25% of genes in human adrenal cortex** | **Chr** | **Position (hg19)** | **Ref base** | **Nonref base** | **dbSNP** | **Freq. NHLBI exomes** | **Freq. 1000 Genomes** | **Impact on protein** |
| --- | --- | --- | --- | --- | --- | --- | --- | --- | --- | --- | --- | --- |
| **CACNA1H** | 5 | 2 | 3.4E-17 | N | chr16 | 1262024 | A | G | Novel | Novel | Novel | M1549V |
|  |  |  |  |  |  |  |  |  |  |  |  |  |

p-value, probability of observing these variants by chance (see below); Top 25% human adrenal cortex expression, expression level according to Ref. 6; Chr, chromosome; Ref / Nonref base, reference / nonreference base; Freq. NHLBI exomes, frequency in NHLBI exome database; Freq. 1000 Genomes, frequency in 1000 Genomes database. Variants were considered previously unreported if absent in dbSNP, NHLBI, 1000Genomes and Yale exome database. The likelihood of observing these variants by chance was calculated as the product of (1) the binomial probability of observing 2 or more *de novo* mutations at a specified position in cases (corrected for the target size of the human exome) and (2) the binomial probability of observing 3 additional independent instances of the identical variant in the remaining 38 subjects (see Materials and Methods).

**Supplementary file 1D.**

**Genes with highest burden of rare (<0.01%) heterozygous variants in cases compared to controls**

| **Gene** | | **# of het carriers among 40 cases** | **# of het carriers among 724 controls** | **p-value** | **Expression in top 25% of genes in human adrenal cortex** | **ID** | | **Chr** | **Position (hg19)** | **Ref base** | **Nonref base** | **dbSNP** | **Freq. NHLBI exomes (%)** | **Freq. 1000 Genomes** | **Impact on protein** |
| --- | --- | --- | --- | --- | --- | --- | --- | --- | --- | --- | --- | --- | --- | --- | --- |
| **LEPREL4** | | 3 | 1 | 5.14E-04 | N |  | |  |  |  |  |  |  |  |  |
|  | |  |  |  |  | 1105-1 | | chr17 | 39965988 | A | T | Novel | Novel | Novel | Y296N |
|  | |  |  |  |  | 1431-1 | | chr17 | 39967425 | C | T | Novel | Novel | Novel | A192T |
|  | |  |  |  |  | 1283-1 | | chr17 | 39967448 | T | C | Novel | Novel | Novel | Y184C |
| **TM7SF3** | | 4 | 5 | 6.41E-04 | Y |  | |  |  |  |  |  |  |  |  |
|  | |  |  |  |  | 1347-1 | | chr12 | 27127128 | C | T | Novel | Novel | Novel | A495T |
|  | |  |  |  |  | 1512-1 | | chr12 | 27143523 | G | A | Novel | Novel | Novel | S243F |
|  | |  |  |  |  | 1390-1 | | chr12 | 27152548 | G | C | Novel | Novel | Novel | P103R |
|  | |  |  |  |  | 110-1 | | chr12 | 27156239 | G | A | Novel | Novel | Novel | S59L |
| **SLC36A1** | | 3 | 2 | 1.24E-03 | N |  | |  |  |  |  |  |  |  |  |
|  | |  |  |  |  | 1506-1 | | chr5 | 150844708 | C | T | Novel | Novel | Novel | R133W |
|  | |  |  |  |  | 1019-1 | | chr5 | 150856227 | C | T | Novel | Novel | Novel | T300I |
|  | |  |  |  |  | 1368-1 | | chr5 | 150867754 | A | C | Novel | Novel | Novel | Y457S |
| **FAM160B1** | | 3 | 2 | 1.24E-03 | Y |  | |  |  |  |  |  |  |  |  |
|  | |  |  |  |  | 270-2 | | chr10 | 116593123 | A | G | Novel | Novel | Novel | K86E |
|  | |  |  |  |  | 333-2 | | chr10 | 116605781 | G | A | rs148193299 | 0.0077 | Novel | V380I |
|  | |  |  |  |  | 1450-1 | | chr10 | 116606018 | G | A | Novel | Novel | Novel | M430I |
| **NPAP1** | | 3 | 3 | 2.39E-03 | N |  | |  |  |  |  |  |  |  |  |
|  | |  |  |  |  | 1390-1 | | chr15 | 24922101 | G | A | rs143596937 | 0.0077 | Novel | E363K |
|  | |  |  |  |  | 270-2 | | chr15 | 24922764 | T | A | rs374388831 | 0.0077 | Novel | S584T |
|  | |  |  |  |  | 1381-1 | | chr15 | 24922968 | C | G | rs367739516 | 0.0077 | Novel | P652A |
| **HNF4G** | | 2 | 0 | 2.68E-03 | N |  | |  |  |  |  |  |  |  |  |
|  | |  |  |  |  | 247-1 | | chr8 | 76452234 | A | G | rs369058537 | 0.0083 | Novel | M3V |
|  | |  |  |  |  | 110-1 | | chr8 | 76471135 | G | A | Novel | Novel | Novel | R319Q |
| **ZMAT3** | | 2 | 0 | 2.68E-03 | N |  | |  |  |  |  |  |  |  |  |
|  | |  |  |  |  | 1452-1 | | chr3 | 178743017 | C | T | Novel | Novel | Novel | Splice, 1 bp upstream of exon 5 |
|  | |  |  |  |  | 110-1 | | chr3 | 178745289 | G | A | rs150264119 | 0.0077 | Novel | R194W |
| **DCAF15** | | 2 | 0 | 2.68E-03 | N |  | |  |  |  |  |  |  |  |  |
|  | |  |  |  |  | 110-1 | | chr19 | 14066728 | G | A | Novel | Novel | Novel | R124Q |
|  | |  |  |  |  | 247-1 | | chr19 | 14070478 | C | T | rs150933337 | 0.0077 | Novel | A435V |
| **MRPL13** | | 2 | 0 | 2.68E-03 | N |  | |  |  |  |  |  |  |  |  |
|  | |  |  |  |  | 1450-1 | | chr8 | 121426252 | C | T | Novel | Novel | Novel | A165T |
|  | |  |  |  |  | 1472-1 | | chr8 | 121457309 | T | C | Novel | Novel | Novel | Q9R |
| **SH3BP1** | | 2 | 0 | 2.68E-03 | N |  | |  |  |  |  |  |  |  |  |
|  | |  |  |  |  | 1390-1 | | chr22 | 38037460 | G | C | Novel | Novel | Novel | S61T |
|  | |  |  |  |  | 1512-1 | | chr22 | 38041459 | T | C | Novel | Novel | Novel | I289T |
| **CEBPG** | | 2 | 0 | 2.68E-03 | N |  | |  |  |  |  |  |  |  |  |
|  | |  |  |  |  | 1325-1 | | chr19 | 33870355 | G | C | Novel | Novel | Novel | E70D |
|  | |  |  |  |  | 1086-1 | | chr19 | 33870542 | G | A | rs200152462 | Novel | Novel | V133I |
|  | |  |  |  |  |  |  | |  |  |  |  |  |  |  |
|  | # of het carriers, number of heterozygous carriers; p-value, result of Fisher's exact test comparing prevalence of variants in cases versus controls; | | | | | | | | | | | | | | |
|  | Top 25% human adrenal cortex expression, expression level according to Ref. 6; ID, subject ID; Chr, chromosome; Ref / Nonref base, reference / nonreference base; | | | | | | | | | | | | | | |
|  | Freq. NHLBI exomes, frequency in NHLBI exome database, Freq. 1000 Genomes, frequency in 1000 Genomes database | | | | | | | | | | | |  |  |  |

**Supplementary file 1E.**

**Genes with highest burden of rare (<0.1%) homozygous, hemizygous or candidate compound heterozygous damaging or conserved variants in cases compared to controls**

| **Gene** | | **# of hom / comp het carriers among 40 cases** | | **# of hom / comp het carriers among 724 controls** | **p-value** | | **Expres-sion in top 25% of genes in human adrenal cortex** | | **ID** | **Het/**  **Hem/Hom var** | **Chr** | **Position (hg19)** | **Ref base** | **Non-ref base** | **dbSNP** | **Freq. NHLBI exomes (%)** | **Freq. 1000 Genomes** | **Impact on protein** |
| --- | --- | --- | --- | --- | --- | --- | --- | --- | --- | --- | --- | --- | --- | --- | --- | --- | --- | --- |
| OR13D1 | | 1 | | 0 | 5.24E-02 | | N | |  |  |  |  |  |  |  |  |  |  |
|  | |  | |  |  | |  | | 1325-1 | Het | chr9 | 107456809 | GA | G | rs368902184 | Novel | het:29, hom:1 | R36fs |
|  | |  | |  |  | |  | | 1325-1 | Het | chr9 | 107457556 | G | GTGAA | rs113720443 | Novel | het:67, hom:3 | G285fs |
| PARP1 | | 1 | | 0 | 5.24E-02 | | N | |  |  |  |  |  |  |  |  |  |  |
|  | |  | |  |  | |  | | 1390-1 | Het | chr1 | 226552729 | G | A | Novel | Novel | Novel | R878W |
|  | |  | |  |  | |  | | 1390-1 | Het | chr1 | 226567724 | C | G | rs138072805 | 0.0231 | het:1,hom:0 | W481S |
| FAM122B | | 1 | | 0 | 5.24E-02 | | N | |  |  |  |  |  |  |  |  |  |  |
|  | |  | |  |  | |  | | 110-1 | Hem | chrX | 133921557 | C | T | Novel | Novel | het:1,hom:0 | V95M |
| PHEX | | 1 | | 0 | 5.24E-02 | | N | |  |  |  |  |  |  |  |  |  |  |
|  | |  | |  |  | |  | | 1024-1 | Hem | chrX | 22132604 | C | T | Novel | 0.0284 | Novel | P401L |
| RBKS | | 1 | | 0 | 5.24E-02 | | Y | |  |  |  |  |  |  |  |  |  |  |
|  | |  | |  |  | |  | | 1543-1 | Hom | chr2 | 28113157 | A | G | rs140995009 | Novel | het:3,hom:0 | V19A |
| MDH1B | | 1 | | 0 | 5.24E-02 | | N | |  |  |  |  |  |  |  |  |  |  |
|  | |  | |  |  | |  | | 1393-1 | Het | chr2 | 207611130 | C | G | rs201274607 | Novel | het:1,hom:0 | G413R |
|  | |  | |  |  | |  | | 1393-1 | Het | chr2 | 207621757 | T | C | rs201485898 | Novel | het:1,hom:0 | Y93C |
| TSPAN6 | | 1 | | 0 | 5.24E-02 | | Y | |  |  |  |  |  |  |  |  |  |  |
|  | |  | |  |  | |  | | 461-1 | Hem | chrX | 99890181 | T | A | Novel | Novel | Novel | R115S |
| DRP2 | | 1 | | 0 | 5.24E-02 | | N | |  |  |  |  |  |  |  |  |  |  |
|  | |  | |  |  | |  | | 1492-1 | Hem | chrX | 100497393 | A | T | Novel | Novel | Novel | D225V |
| SRPX2 | | 1 | | 0 | 5.24E-02 | | Y | |  |  |  |  |  |  |  |  |  |  |
|  | |  | |  |  | |  | | 1147-1 | Hem | chrX | 99917183 | G | C | Novel | Novel | Novel | W58C |
| KALRN | | 1 | | 0 | 5.24E-02 | | Y | |  |  |  |  |  |  |  |  |  |  |
|  | |  | |  |  | |  | | 771-1 | Het | chr3 | 124385344 | G | A | rs201076232 | Novel | Novel | V2131M |
|  | |  | |  |  | |  | | 771-1 | Het | chr3 | 124418784 | C | T | rs200600368 | Novel | Novel | P2634S |
| MAP3K15 | | 1 | | 0 | 5.24E-02 | | Y | |  |  |  |  |  |  |  |  |  |  |
|  | |  | |  |  | |  | | 1450-1 | Hem | chrX | 19506985 | C | A | Novel | 0.0246 | Novel | L154F |
| NRK | | 1 | | 0 | 5.24E-02 | | Y | |  |  |  |  |  |  |  |  |  |  |
|  | |  | |  |  | |  | | 1543-1 | Hem | chrX | 105156700 | C | G | Novel | 0.0955 | Novel | Q768E |
|  | |  | | |  | |  |  |  |  |  |  |  |  |  |  |  |  |

# of hom / compound het carriers, number of homozygous or candidate compound heterozygous carriers; p-value, result of Fisher's exact test comparing prevalence of variants in cases versus controls; Top 25% human adrenal cortex expression, expression level according to Ref. 6; ID, subject ID; Het/Hem/Hom var, heterozygous, hemizygous or homozygous variant; Indep ref / nonref reads, independent reference / nonreference reads; Chr, chromosome; Ref / Nonref base, reference / nonreference base; NHLBI exomes, frequency in NHLBI exome database; 1000 Genomes, frequency in 1000 Genomes database; AA change, amino acid change; fs, frameshift

**Supplementary file 1F.**

**Illumina sequence reads identifying CACNA1H p.Met1549Val in 5 unrelated subjects**

| **Sample ID** | **Chr** | **Gene** | **Position (hg19)** | **Ref base** | **Nonref base** | **Indep ref reads** | **Indep nonref reads** | **Quality score** | **Het/Hom var** | **AA change** | **Control Freq** |
| --- | --- | --- | --- | --- | --- | --- | --- | --- | --- | --- | --- |
| 1347-1 | chr16 | *CACNA1H* | 1262024 | A | G | 58 | 48 | 228 | Het | M1549V | 0 |
| 1368-1 | chr16 | *CACNA1H* | 1262024 | A | G | 78 | 49 | 228 | Het | M1549V | 0 |
| 1390-1 | chr16 | *CACNA1H* | 1262024 | A | G | 71 | 64 | 228 | Het | M1549V | 0 |
| 1393-1 | chr16 | *CACNA1H* | 1262024 | A | G | 158 | 85 | 228 | Het | M1549V | 0 |
| 333-2 | chr16 | *CACNA1H* | 1262024 | A | G | 59 | 55 | 228 | Het | M1549V | 0 |

Chr, chromosome; Ref, reference; Nonref, non-reference; Indep ref reads, number of independent reference reads; Quality score, SAMtools quality score (maximum 228); Het/Hom var, heterozygous or homozygous variant inferred; AA change, amino acid change from reference; Control frequency, # of times variant called among >129,000 alleles in Exome Aggregation Consortium database (Exome Aggregation Consortium, 2014) and Yale databases.

**Supplementary file 1G.**

**Demonstration of biological parentage by genotyping of short tandem repeat markers in parent-offspring trios in kindreds 1347 and 1390 confirms that *CACNA1H* mutations are *de novo* in these kindreds**

| **Locus**  **Individual** | | **CSF1PO** | **D13S317** | **D16S539** | **D18S51** | **D7S820** | **D8S1179** | **THO1** | **TPOX** | **D19S433** | **CPI/CMI** |
| --- | --- | --- | --- | --- | --- | --- | --- | --- | --- | --- | --- |
| **1347-1**  **(Proband)** | **Genotype** | 11,12  (0.30, 0.36) | 13,13  (0.12, 0.12) | 12,12  (0.33, 0.33) | 14/18  (0.14, 0.08) | 10,12  (0.24, 0.17) | 11,11  (0.08, 0.08) | 3,10  (NA, 0.01) | 11,11  (0.24, 0.24) | 13,14  (0.25, 0.37) |  |
| **1347-2**  **(Mother)** | **Genotype** | 12,12  (0.36, 0.36) | 12,13  (0.25, 0.12) | 11,12  (0.32, 0.33) | 12,18  (0.13, 0.08) | 11,12  (0.21, 0.17) | 11,12  (0.08, 0.19) | 3,7  (NA, 0.19) | 8,11  (0.53, 0.24) | 14,15  (0.37, 0.15) |  |
|  | **MI** | 1.39 | 4.03 | 1.53 | 3.28 | 1.51 | 6.04 | NA | 2.05 | 0.68 | **356.15** |
| **1347-3**  **(Father)** | **Genotype** | 11,12  (0.30, 0.36) | 12,13  (0.25, 0.12) | 12,14  (0.33, 0.02) | 14,16  (0.14, 0.14) | 8,10  (0.15, 0.24) | 11,11  (0.08, 0.08) | 6,10  (0.23, 0.01) | 8,11  (0.53, 0.24) | 13,14  (0.25, 0.37) |  |
|  | **PI** | 1.52 | 4.03 | 1.53 | 1.82 | 1.03 | 12.08 | 30.19 | 2.05 | 0.99 | **12986.91** |
| **1390-2**  **(Proband)** | **Genotype** | 10,12  (0.26, 0.30) | 11,12  (0.31, 0.42) | 11,12  (0.32, 0.20) | 17/19  (0.15, 0.10) | 8,10  (0.24, 0.33) | 10,12  (0.03, 0.14) | 6,7  (0.12, 0.42) | NA | 10,12  (0.01, 0.11) |  |
| **1390-5**  **(Mother)** | **Genotype** | 7,10  (0.05, 0.26) | 12,13  (0.42, 0.15) | 11,12  (0.32, 0.20) | 15,17  (0.16, 0.15) | 8,12  (0.24, 0.09) | 10,12  (0.03, 0.14) | 7,7  (0.42, 0.42) | NA | 12,13  (0.11, 0.25) |  |
|  | **MI** | 0.97 | 0.59 | 2.06 | 1.65 | 1.06 | 10.37 | 1.19 | NA | 2.19 | **55.56** |
| **1390-10**  **(Father)** | **Genotype** | 11,12  (0.25, 0.30) | 11,12  (0.31, 0.42) | 11,11  (0.32, 0.32) | 18,19  (0.12, 0.10) | 9,10  (0.11, 0.33) | 12,12  (0.14, 0.14) | 6,7  (0.12, 0.42) | NA | 10,11  (0.01, 0.06) |  |
|  | **PI** | 0.84 | 1.41 | 1.57 | 2.52 | 0.75 | 3.53 | 2.61 | NA | 25.80 | **840.08** |

Genotypes at indicated highly polymorphic marker loci in parent-offspring trios are shown. Estimated ethnicity-specific allele frequencies are shown below genotypes in parentheses (Butler et al., 2003). PI/MI denotes paternity/maternity index (Brenner et al., 1993) (Materials and methods). CPI/CMI, combined paternity/maternity index. NA, not available. The CPI is a likelihood ratio comparing the likelihood of the observed results under the alternative hypotheses that the alleged parent is a biological parent vs. an unrelated person of similar ancestry. No trio genotypes were inconsistent with both purported biological parents being true biological parents.

**Supplementary file 1H.**

**Kinship coefficients of affected individuals from kindreds with *CACNA1H^M1549V^* variant**

| **ID1** | **ID2** | **Kinship coefficient** |
| --- | --- | --- |
| 1347-1 (CEU) | 1368-1 (CEU) | -0.0562 |
| 1347-1 (CEU) | 1390-1 (AA) | -0.2518 |
| 1347-1 (CEU) | 1393-1 (HIS) | -0.0880 |
| 1347-1 (CEU) | 333-1 (CEU) | -0.0499 |
| 1368-1 (CEU) | 1390-1 (AA) | -0.2567 |
| 1368-1 (CEU) | 1393-1 (HIS) | -0.0946 |
| 1368-1 (CEU) | 333-1 (CEU) | -0.0577 |
| 1390-1 (AA) | 1393-1 (HIS) | -0.1549 |
| 1390-1 (AA) | 333-1 (CEU) | -0.2415 |
| 1393-1 (HIS) | 333-1 (CEU) | -0.0838 |

Affected subjects from five kindreds with the *CACNA1H^M1549V^* variant were genotyped on Illumina Human 1M beadchips, and all pairwise kinship coefficients were determined using the robust algorithm in KING 1.4. Negative kinship coefficients indicate unrelated relationships, and very negative values are suggestive of individuals from different populations (Manichaikul et al., 2010). CEU, of European ancestry; AA, of African American ancestry; HIS, of Hispanic ancestry.

**Supplementary file 1I.**

**Clinical features of family members of index cases with *CACNA1H^M1549V^***

| **Subject ID** | **Gender** | **Age dx / eval*** | **BP**  (%ile) | **Aldo**  (ng/dL) | **PRA**  (ng/mL/h) | **ARR (Aldo/PRA)** | **Direct renin** (μIU/mL) | **Aldo/**  **direct renin** |
| --- | --- | --- | --- | --- | --- | --- | --- | --- |
| **333-1** | M | 5 yrs | 160/120 (>99th) | NA | NA | NA | NA | NA |
| **1390-2** | F | 17 yrs | 215/115 (>99th) | 22 | NA | NA | 3 | 7.3 |
| **1393-3** | M | 38 yrs* | 120/82 (>50th) | 9.3 | 0.81 | 11.5 | NA | NA |
| **1368-2** | F | 49 yrs* | 116/80 (>25th) | 8.0 | 4.73 | 1.7 | NA | NA |
| **1368-4** | M | 24 yrs | 200/100 (>95th) | 11.0** | 1.2** | 9.2** | NA | NA |

M, male; F, female; age dx, age at diagnosis of hypertension; eval*, age at evaluation in the absence of hypertension; yrs, years; BP, blood pressure; (%ile), percentile adjusted for age and gender; Aldo, serum aldosterone; PRA, plasma renin activity; ARR (Aldo/PRA), aldosterone:renin ratio using plasma renin activity (values >20 indicative of primary aldosteronism); Direct renin, values <5 suggestive of volume expansion-mediated hypertension; Aldo/direct renin, values >2.4 with aldosterone level greater than 15 considered indicative of primary aldosteronism; **, values measured while taking hydrochlorothiazide; NA, not available.

**References**

Brenner CH. 1993. A note on paternity computation in cases lacking a mother. *Transfusion* **33**:51-4. doi: [10.1046/j.1537-2995.1993.33193142310.x](http://dx.doi.org/10.1046/j.1537-2995.1993.33193142310.x).

Butler JM, Schoske R, Vallone PM, Redman JW, Kline MC. 2003. Allele frequencies for 15 autosomal STR loci on U.S. Caucasian, African American, and Hispanic populations. *Journal of Forensic Sciences* **48**:908-11.

Exome Aggregation Consortium (ExAC). 2014. Cambridge, MA (URL: <http://exac.broadinstitute.org)>. [accessed Dec 2014].

Manichaikul A, Mychaleckyj JC, Rich SS, Daly K, Sale M, Chen WM. 2010. Robust relationship inference in genome-wide association studies. *Bioinformatics* **26**:2867-73. doi: [10.1093/bioinformatics/btq559](http://dx.doi.org/10.1093/bioinformatics/btq559).
